# Tau Ubiquitination in Alzheimer's Disease

**DOI:** 10.3389/fneur.2021.786353

**Published:** 2022-02-08

**Authors:** Longfei Li, Yanli Jiang, Jian-Zhi Wang, Rong Liu, Xiaochuan Wang

**Affiliations:** ^1^Key Laboratory of Education Ministry/Hubei Province of China for Neurological Disorders, Department of Pathophysiology, School of Basic Medicine, Tongji Medical College, Huazhong University of Science and Technology, Wuhan, China; ^2^Co-innovation Center of Neuroregeneration, Nantong University, Nantong, China; ^3^Department of Pathology and Pathophysiology, School of Medicine, Jianghan University, Wuhan, China

**Keywords:** PHF, Alzheimer's disease, tau, NGF, ubiquitination

## Abstract

Paired helical filaments (PHFs) from the Alzheimer's disease (AD) brain are highly ubiquitinated and ubiquitination likely plays a vital role in tau filament formation. Whether tau ubiquitination is the causality or consequence of the disease in AD remains elusive. The following questions are worth considering: What does the extent of tau ubiquitination contribute to tau pathology in AD? Does tau ubiquitination influence aggregation or spreading during disease progression? In addition, tau is polyubiquitinated in nerve growth factor-induced PC12 cells and participates in mitogen-activated protein kinase signaling, in addition to its microtubule stabilization function. Therefore, ubiquitination possibly mediates tau signaling under physiological conditions, but tau aggregation in the pathobiology of AD. Here, we review the advancements in tau ubiquitination and the potential therapeutic effects of targeting tau ubiquitination to alleviate tau pathology in AD.

## Introduction

Ubiquitin-positive inclusions are characteristic of neurodegenerative diseases, such as Alzheimer's disease (AD) and frontal temporal lobe degeneration (1–3). Neurofibrillary tangles, mainly consisting of hyperphosphorylated tau, are also ubiquitin and p62 positive in AD (4, 5). In fact, ubiquitin is elevated by many folds in the AD brain determined by immunoassay (1), and the ubiquitination level increases at ~80% ubiquitylated sites from the altered 800 ubiquitination sites by label-free mass spectrometry (MS)-based proteomic analysis (6). Among the ubiquitination proteins, the microtubule-associated protein tau has the highest number of sites of ubiquitination per protein in AD (6). In addition, the data from cryoelectron microscopy (EM) and MS of tau filaments from AD and corticobasal degeneration brain further demonstrate ubiquitination of tau in neurofibrillary tangles, and this posttranslational modification might play a structural role in its fibrillation and fibril heterogeneity (7–9). This review aimed to conclude the influence of tau ubiquitination in the pathobiology of AD and provide valuable cues for future studies.

## Type Of Tau UBIQUITINATION In Ad

The 76-amino acid protein ubiquitin can be transferred to lysine with subjects through the three enzyme cascade steps: ubiquitin activation, conjugation, and ligation by E1, E2, and E3 enzymes in succession (10). The substrate can be either modified by a monomer or a polyubiquitin chain depending on E2s, whereas the substrate specificity is determined by E3 ligases (11). In addition to monoubiquitylation, a polyubiquitin chain occurs when another ubiquitin is conjugated to any of the seven lysines or N-terminus on the first ubiquitin by its C-terminal GG amino acid (12). Ubiquitin modification and its signal for cell output and degradation are reviewed in detail (10–14). In this review, these were not specified, and this review only focused on tau ubiquitination in AD.

**Figure 1:**
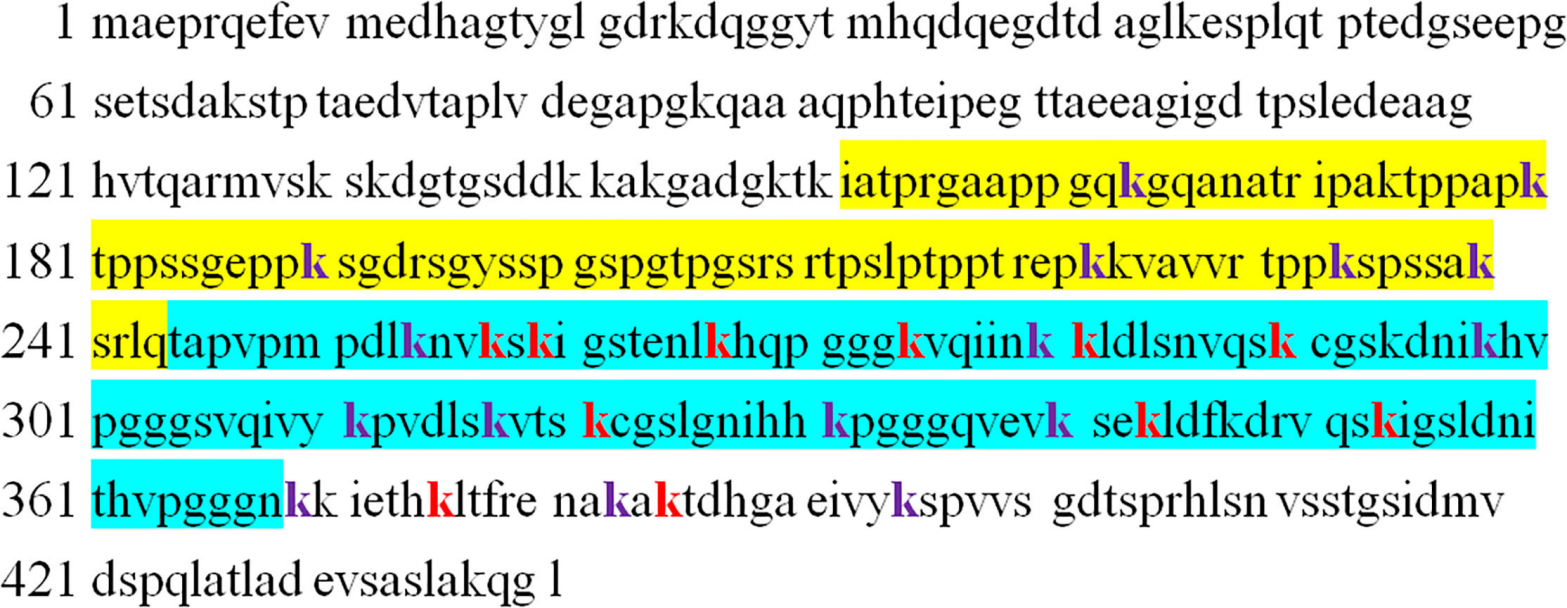
The sequence of 2N4R-tau with identified ubiquitinated sites (both purple and red, k). The yellow sequence indicates proline-rich domain, whereas cyan indicates microtubule-binding repeat domain. Chip ubiquitinates tau at sites shown by red color.

The microtubule-associated protein tau has 44 lysine residues on the longest 441 isoforms, most of which are located on the proline-rich domain and microtubule-binding domain repeat (MTBR) (15). Tyrosine kinase family proteins, such as fyn and c-src, bind to tau with the PXXP motif in the proline-rich domain and execute its function (16, 17), whereas tau promotes microtubule stabilization through the MTBR domain (18). The abundant lysine and arginine make full-length tau pI = 8.24, and tau's repeat domain (termed K18) pI = 9.73 (15). Furthermore, 28 tau ubiquitylation sites detected in human AD brain samples underlie the tau to become the highest number of increased ubiquitination sites per protein, as discussed above (6). Among these sites, K257, K259, K267, K274, K281, K290, K321, K343, K353, K375, and K385 are ubiquitinated by E3 ligase Chip (Figure 1) (7). The most frequently reported sites are Lys254, Lys257, Lys311, Lys317, and Lys353 (6, 8, 19, 20).

To date, K48-, K63-, K6-, K11-, and M1-linked polyubiquitin chains have been verified in tau of paired helical filament (PHF), although the majority of ubiquitination in PHF is in monoubiquitinated form (19–21). K48-linked polyubiquitination is the most common form of tau poly-Ub and is hypothesized to mediate protein degradation by the proteasome system (19). K63-linked poly-Ub of proteins can serve multiple functions for various proteins, including promoting insoluble inclusion formation (22), signal for autophagy lysosome (13), endocytosis (23), and DNA repair (24). A part of PHF is K63-linked poly Ub-positive, as detected in the AD brain (25). One study reported that to a lesser extent K6/11 poly-Ub existed in PHF by tandem mass spectrometry assay, and K6-linked poly-Ub may inhibit proteasome activity (20). M1-linked linear poly-Ub, which is involved in neuronal cell death, appears after K48-linked poly-Ub of PHF (21, 26). A few studies revealed that tau mono-Ub impaired its microtubule binding (27) and that the N-terminal tau mono-Ub could impede its aggregation by cooperating with the proteasome system (28).

## Regulation Of Tau UBIQUITINATION In Ad

The identification of the types of tau ubiquitination is accompanied by the recognition of the E1, E2, and E3 enzymes involved in tau ubiquitination. Regarding mono-Ub, axotrophin/MARCH7 containing a RING-variant domain could monoubiquitinate tau combined with E2 UbcH5 and impair its microtubule binding (29). A recent study reported that E2 Ube2W attached mono-Ub to the αN-terminus of tau by recognizing backbone atoms of disordered N-termini (30). *In vivo*, CHIP modifies tau through both K48- and K63-linked polyubiquitination (31), and tau is also a K63-polyubiquitinated substrate of TRAF6/UbcH7 (32). Furthermore, the ubiquitin elongation enzyme UBE4B has been reported to promote tau polyubiquitination sufficiently by cooperating with CHIP, thereby promoting tau degradation (33).

In addition to identifying E3 ligase on tau ubiquitination, other inducers, such as nerve growth factor (NGF), can promote tau ubiquitination through unknown mechanisms. NGF maintains neuronal survival and differentiation in PC12 through its receptor TrkA with the elevation (>2-fold) of ubiquitin conjugate levels; a similar result could be mimicked by a proteasome inhibitor (34). NGF also stimulates tau ubiquitination through K63-linked poly-Ub in neuronal cell differentiation (25), which implies the possibility of the downregulation of the NGF signaling pathway in AD pathogenesis by impairing normal tau ubiquitination, as the decrease of NGF in the cortex and hippocampus mediates cholinergic neuron degeneration in the basal forebrain at disease onset.

## Role Of UBIQUITINATION In Tau Turnover

Six isoforms of tau by alternative slicing are mainly expressed in the central nervous system (CNS) (35). CNS tau is a long-lived and natively disordered protein, which has a half-life of ~23 days. Under physiological conditions, tau is soluble and has a hairpin structure in which the MTBR is buried under the inner layers of the C-terminus and the outer layers of the N-terminus, as detected by fluorescence resonance energy transfer (36). An average of 2–3 mol of phosphate per mole of protein in normal people and 6–8 mol of phosphate per mole of protein in the AD brain indicates that abnormal hyperphosphorylation of tau plays an important role in the pathobiology of AD (37–39). Several studies have already reported how hyperphosphorylation of tau leads to neurotoxicity and epigenetic risk factors, such as trauma, sleeplessness, and less exercise, influencing tau phosphorylation and cognitive function (40). Thus, tau phosphorylation is widely used as a biomarker in cell and animal models in AD studies. Tau phosphorylation at threonine 181 and 217 in the cerebrospinal fluid or plasma have shown their potential as a biomarker for predicting the extent of dementia (41, 42). AT8 staining is considered the gold standard for detecting pretangles in immunohistochemistry (IHC), as it is applied by BraaK (43).

Phosphorylation of tau could change its conformation and open its hairpin structure, resulting in the formation of tau oligomers despite the exact mechanisms of oligomer formation being still elusive. Many factors can affect this process, such as extending its N-terminus or C-terminus to protease, resulting in truncation and accelerating tau aggregation, or being recruited to stress granules with its abundant lysine in the proline-rich (PR) and MTBR domains under cell stress (15, 44–47). In 1989–1991, it was reported that ubiquitin levels increased many folds in the AD brain, and the accumulation of abnormally phosphorylated tau preceded the formation and ubiquitination of neurofibrillary tangle (NFT) (1, 48, 49). About 2 years later, Yasuo Ihara et al. purified high-molecular weight (HMW) Ub (–) PHF and ubiquitin (+) PHF from PHF tau and found that the amino-terminal portion of tau was truncated in Ub (–) PHF and to a greater extent in HMW ubiquitin (+) PHF (19). A more detailed study was conducted several years later, using tau oligomer-specific antibody T22 in combination with pT231, AT8, and ubiquitin antibodies. Kayed et al. proposed a more specific sequence of tangle formation with minor revision of previous results: tau phosphorylation at the T231 site (stage 0); tau oligomer initiation and formation with pT231 (stage 1) and intraneuronal NFT (iNFT) containing mixed oligomer, protofilament, and filament (stage 2); and neuronal death and the formation of ghost tangle with T22 negativity and pT231 positivity, referred to as extraneuronal NFT (eNFT) (stage 3). Besides the AT8 property of the staining pretangle, most eNFTs are AT8 positive. By comparing the time sequence of ubiquitination and tau oligomerization, they suggest that ubiquitination does not appear at the initial stage of tau oligomerization, but it occurs on iNFT (stage 2) followed by high ubiquitination on eNFT (50). The above studies suggest that abnormal phosphorylation likely mediates tau oligomerization (51). As the soluble tau oligomer grows and is modified by truncation and ubiquitination, it gradually matures to fibrillary tangles and is insoluble with tangle formation. This transformation suggests that ubiquitination probably promotes insoluble filament formation from soluble tau aggregates (8, 52).

To better understand the effect of ubiquitination on tau aggregation, Kah-Leong Lim explored the contribution of K48- and K63-linked poly-Ub and monoubiquitination to the biogenesis of inclusions in cell models and found that K63-linked poly-Ub usually promoted inclusion formation, such as tau and sod1 aggregates and their clearance by autophagy (22). In fact, CHIP-mediated K48-/K63-linked poly-Ub promotes insoluble tau formation, whereas HSP70 suppresses it (31). Moreover, CHIP knockout mice show accumulation of nonaggregated, ubiquitin-negative, and hyperphosphorylated tau, demonstrating that CHIP mediates ubiquitin-dependent tau degradation and poly-Ub of tau by CHIP accelerating the formation of insoluble filaments (53). CHIP is an E3 ligase of phosphorylated tau. CHIP mediates tau ubiquitination at K267, K290, K343, and K353 sites, but additional K257, K259, K274, K281, K321, K375, and K385 are ubiquitinated by CHIP after tau is phosphorylated by glycogen synthase kinase-3β. Prolonged phosphorylated tau ubiquitination promotes tau aggregation (7). In another study, traf6-mediated K63-linked poly-Ub also showed a similar effect on tau aggregation (32).

Contrary to these studies, Munari et al. recently studied the aggregation rate of K18 and Ub-K18 *in vitro* by applying semisynthetic and enzyme-mediated conjugate methods (27). They used CHIP/UBC13 rather than E2 UBCH5 to perform K18 (fragment of 4R-tau) ubiquitination in the tube, which resulted in tau mono-Ub rather than poly-Ub. Unexpectedly, these modifications make K18 lose the ability to convert into amyloid fibrillary structures. Meanwhile, conjugating the mono-Ub to a specific site in K18 by the semisynthetic method would slow down the aggregation but permit the aggregation formation at Lys254 and mostly at Lys353. Lys 311 ubiquitination might follow the formation of NFT and determine fibril subtypes because it is located within the core region of tau for the formation of NFT, which will hinder the aggregation (27).

The N-terminal connected mono-Ub of K18 by UBE2W also slows down the K18 aggregated kinetics and targets oligomers formed by N-mono-Ub-modified K18 to proteasomal degradation compared to unmodified K18 (28). These studies suggest that ubiquitination influences tau aggregation *in vitro*. However, it does not consider which ubiquitination usually occurs later than tau phosphorylation and oligomerization, as discussed above, in pathological conditions, although tau monomer needs ubiquitination to be degraded by proteasomes in physiological status.

Proteasomes have recently been shown to fragment tau filaments into oligomers *in vitro* and degrade oligomers formed from N-terminal mono-Ub-linked K18 (54). Regardless of whether CHIP or Traf6 mediates tau ubiquitination, both have been demonstrated to promote tau degradation by the proteasome. In contrast, tau oligomers can impair proteasomes and decrease proteasome activity in AD brains (55). Therefore, the following should be taken into consideration: (1) Elevation of ubiquitin several folds in the AD brain and ubiquitin-positive inclusions being common in neurodegeneration; (2) the spatiotemporal order of tau phosphorylation, oligomerization, and ubiquitination during the formation of PHF; (3) loss of proteasome activity in the AD brain (56); and (4) the neurotoxicity of oligomer tau rather than PHF (57, 58). This can outline the following vicious circle model in cells to explain the roles of ubiquitination in the regulation of tau degradation and aggregation: (1) protein folding-refolding machinery (CHIP/HSP70/90) supervises the folding status of tau (normal tau stage); (2) once tau is abnormally phosphorylated with conformational changes and forms oligomers, a protein quality control system will observe these changes and initiate ubiquitination of the misfolded protein, which promotes insoluble tau formation and its degradation by the proteasome (oligomer tau stage); and (3) with consistent oligomer tau stages, the proteasome is overloaded and cracked; however, without the normal function of the proteasome, these insoluble aggregates accumulate over time and mature into filaments with further ubiquitination, phosphorylation, and truncation (Ub + PHF) (NFT stage).

## Is Tau UBIQUITINATION Beneficial To Neuron Survival In Ad?

**Figure 2:**
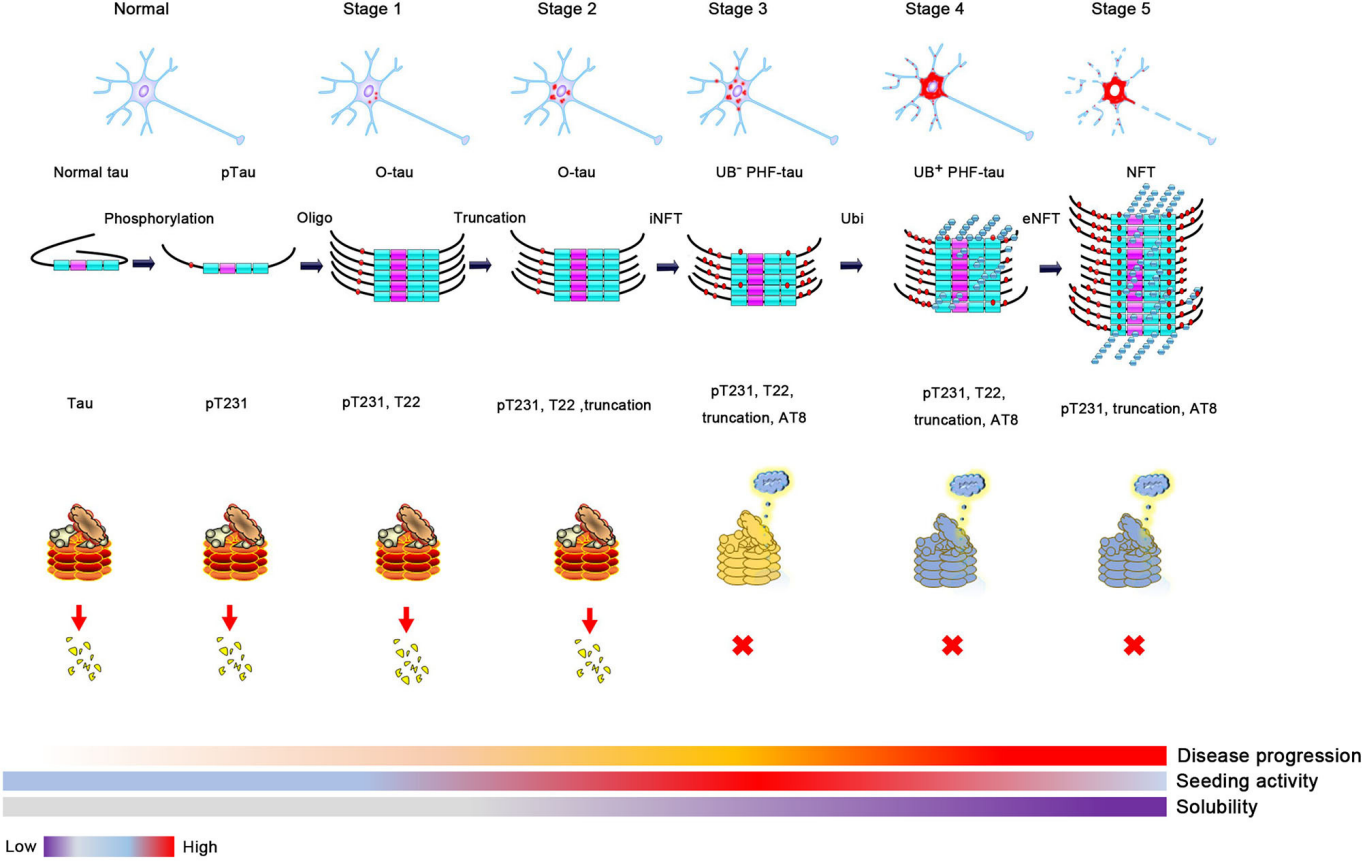
The proposed process of tau aggregation. Phosphorylation changes tau conformation and induces oligomer tau aggregation. Soluble oligomer tau has higher toxicity and seeding activity. Proteases, such as caspase 3, calpain, or legumain, can cleavage tau and result in tau truncation when the cell is toxic. Monomer and oligomer tau can be ubiquitinated and degraded by the proteasome. However, ubiquitin-positive tau aggregation will be accumulated when the activity of proteasome is decreased (high activity, red; medial activity, yellow; and low activity, blue), which will decrease the seeding activity and solubility of tau aggregation and finally lead to the formation of neurofibrillary tangles formation.

Despite evidence suggesting that tau ubiquitination is involved in tau pathology, including tau mislocated to dendrites or promoting tau aggregation (7, 59), there is no direct evidence showing that tau ubiquitination is neurotoxic. However, as the primary function of ubiquitination is to mediate protein degradation, tau ubiquitination is likely nontoxic. Hyperphosphorylated soluble and oligomer tau is hypothesized to have the highest seeding activity and is the most toxic tau species among tau monomers, oligomers, and multimers (58, 60–63). As discussed above, ubiquitination increases tau insolubility and occurs at a relatively late stage during the formation of NFTs. Thus, soluble and toxic tau oligomers are transformed into insoluble and less toxic NFTs after hyperubiquitination. In fact, neurons bearing NFTs can survive for many years (64, 65). Soluble tau, rather than insoluble NFTs, results in neuronal loss and cognitive dysfunction in a model of tauopathy (57). Based on this evidence, ubiquitination likely protects neurons from the toxicity of oligomer tau by promoting insoluble NFT formation, which is consistent with the concept that polyubiquitination promotes tau aggregation. In contrast, the prion-like activity of pathologic tau decreases with longevity, despite an increase in insoluble tau in AD, and the seeding activity of oligomer tau is higher than that of sarkosyl-insoluble tau. Thus, we can hypothesize that ubiquitination will decrease the seeding and toxic activity of oligomer tau by promoting insoluble NFT formation, but this needs to be proven by experiments. Under physiological conditions, the tau monomer or oligomer is ubiquitinated and mediated to the proteasome for degradation. However, as AD progresses, proteasome impairment results in the accumulation of ubiquitinated proteins. The dysfunction of this process might contribute to the formation of NFTs and result in neurofibrillary neurodegeneration (Figure 2).

## Alzheimer'S Disease Therapeutic Approaches Based On Tau UBIQUITINATION

As discussed above, ubiquitination is good for tau degradation or promotes insoluble and less-toxic aggregate formation from soluble toxic oligomers under physiological or pathological conditions. Therefore, targeting tau ubiquitination should be an effective approach for the treatment of AD. In fact, the benefits of tau ubiquitination are supported by the fact that upregulation of tau ubiquitination through biochemical methods can reduce tau levels and improve impaired memory in an AD mouse model *in vivo*. Using a chimera with tau binding domain and E3 ligase-binding moieties, TH006 can specifically induce endogenous tau degradation by promoting tau polyubiquitination (66). A similar chimera, C004019, with the same routine has similar effects and improves cognitive function in an AD mouse model (67). In addition, mislocated tau in the postsynaptic fraction is hyperphosphorylated and ubiquitinated, and tau from the postsynaptic fraction is a seeding component. Promoting tau degradation in this area by elevating proteasome activity by stimulating the PAC1 receptor-mediated cyclic adenosine monophosphate/protein kinase A (cAMP/PKA) pathway has been shown to improve impaired cognitive performance in rTg4510 mice (67). Another direct evidence is from the Drosophila transgenic model. Overexpression of UBE4B, an E4 ubiquitin elongation enzyme, promotes tau ubiquitination and degradation and alleviates eye neurodegeneration, which is blocked by knockout of the E3 ligase CHIP. Furthermore, this enzyme can reduce tau oligomer levels in a tau transgenic model (33). Based on this evidence, we can conclude that promoting tau ubiquitination or elevating proteasome activity promotes tau degradation and improves tau-induced neurodegeneration and cognitive dysfunction *in vivo*, despite the fact that hyperpolyubiquitination promotes tau aggregation *in vitro*. Therefore, promoting tau ubiquitination along with elevated proteasome activity might provide an effective avenue for AD treatment by reducing tau protein levels.

## Conclusion And Remarks

Ubiquitination can induce ubiquitin-positive and ubiquitin-insoluble tau formation, which could mediate protein degradation. Polyubiquitination protects cells from the toxicity of the soluble oligomer tau. However, these ubiquitin-positive and insoluble tau aggregates accumulate in cells if the degradation system is damaged under disease conditions. Ubiquitination of tau is good for cell survival, as many studies aimed to induce tau ubiquitination artificially, having beneficial effects on improving abnormal behavior in an AD mouse model. In addition, the genetic upregulation of E3 or E4 ubiquitin ligases is also useful. Among them, CHIP is the most well-studied E3 ligase for tau ubiquitination. CHIP mainly ubiquitinates phosphorylated tau and promotes the formation and degradation of insoluble tau. Deletion of CHIP in P301L mice led to soluble and phosphorylated tau accumulation. Furthermore, the sites in tau ubiquitinated by CHIP have recently been identified *in vitro* (7). These studies strongly suggest that ubiquitination promotes NFT formation. In other words, NFT formation might be a consequence of tau ubiquitination without being efficiently degraded by the proteasome. Thus, promoting proteasome activity degrades these ubiquitin-positive tau aggregates. In contrast, reducing tau ubiquitination by inactivating E3 ligase results in soluble tau accumulation, which is a disaster for cell survival. Because HMW tau oligomers in the soluble fraction have higher seeding activity and are the most toxic, it is worthwhile to study the effect of ubiquitination on oligomer tau seeding activity and neurotoxic ability. As phosphorylation or acetylation of tau has been widely explored, the role of ubiquitination in tau-mediated neurodegeneration is believed to be the next hot topic in the future.

## Author Contributions

XW framed and reviewed the manuscript. LL and YJ organized the literature and wrote the manuscript. J-ZW and RL analyzed and discussed the manuscript. All the authors read and approved the final version of the manuscript.

## Funding

This review article was supported by grants from the National Natural Science Foundation of China (92049107, 31771114, and 31929002), the Innovative Research Groups of the National Natural Science Foundation of China (81721005), and the Academic Frontier Youth Team Project to XW from the Huazhong University of Science and Technology.

## Conflict of Interest

The authors declare that the research was conducted in the absence of any commercial or financial relationships that could be construed as a potential conflict of interest.

## Publisher's Note

All claims expressed in this article are solely those of the authors and do not necessarily represent those of their affiliated organizations, or those of the publisher, the editors and the reviewers. Any product that may be evaluated in this article, or claim that may be made by its manufacturer, is not guaranteed or endorsed by the publisher.
